# Seizures Risk Related to Atypical Antipsychotics: A Disproportionality Analysis Based on the FDA Adverse Event Reporting System (FAERS)

**DOI:** 10.31083/AP44501

**Published:** 2026-04-21

**Authors:** Xue Zhao, Yu Cheng, Shen He, Fan Mou, Wei Li, Shunying Yu

**Affiliations:** ^1^Shanghai Mental Health Center, Shanghai Jiao Tong University School of Medicine, 200030 Shanghai, China

**Keywords:** antipsychotic agents, seizures, pharmacovigilance, FAERS, disproportionality analysis

## Abstract

**Background:**

This pharmacovigilance study aimed to characterize the spectrum of seizure-related adverse events (AEs) associated with eight commonly used atypical antipsychotics (AAPs)—clozapine, quetiapine, olanzapine, aripiprazole, ziprasidone, risperidone, lurasidone, and paliperidone—based on the Food and Drug Administration (FDA) Adverse Event Reporting System (FAERS) database, and to explore potential receptor mechanisms underlying AAP-related seizures.

**Methods:**

Disproportionality analysis was performed using FAERS data from the first quarter of 2004 to the second quarter of 2025. Signal values were assessed using the Reporting Odds Ratio (ROR), Proportional Reporting Ratio (PRR), Bayesian Confidence Propagation Neural Network (BCPNN), and Multi-item Gamma Poisson Shrinker (MGPS) algorithms. Spearman correlation analysis was performed to examine associations between ROR values and receptor binding affinity (Ki) data for D1, D2, D3, 5-HT_1A_, 5-HT_2A_, 5-HT_2C_ receptors and 5-HT_1A_/D2, 5-HT_2A_/D2, and 5-HT_2C_/D2 receptor ratios.

**Results:**

The final analysis included 9057 reports of AAPs–seizures pairs (N ≥3), and 8540 cases demonstrating significant seizures-related signals detected by ROR analysis at the preferred term (PT) level. The eight AAPs demonstrated distinct seizures AE profiles, with quetiapine associated with the broadest spectrum (15 PTs). At the High-Level Group Term (HLGT) level, ziprasidone showing the strongest association (ROR_025_ = 3.21), followed by quetiapine (ROR_025_ = 2.62), clozapine (ROR_025_ = 2.58), olanzapine (ROR_025_ = 2.53) and aripiprazole (ROR_025_ = 2.05). Risperidone, paliperidone, and lurasidone exhibited comparatively lower signals. Spearman correlation analysis revealed a significant negative correlation between the 5-HT_1A_/D2 receptor affinity ratio and ROR values (rs = –0.79, *p* = 0.036).

**Conclusions:**

This large-scale real-world analysis suggests that clozapine, quetiapine, olanzapine, aripiprazole, and particularly ziprasidone are associated with higher reporting signals for seizures compared with risperidone, paliperidone, and lurasidone. The inverse correlation with the 5-HT_1A_/D2 ratio suggests a potential pharmacodynamic mechanism. Clinicians should consider these differential risks, especially when prescribing for patients with predisposing factors, and maintain vigilance for specific seizures types. These findings warrant further validation through prospective studies and clinical causality assessments.

## Main Points

1. Seizures are a rare but serious adverse reaction associated with antipsychotic treatment and warrant clinical vigilance during drug selection.

2. Compared with typical antipsychotics, atypical antipsychotics are associated with lower rates of some adverse effects, such as extrapyramidal symptoms, but may carry a higher risk of seizures.

3. Food and Drug Administration (FDA) Adverse Event Reporting System (FAERS) analysis suggested that, compared with risperidone, paliperidone, and lurasidone, clozapine, quetiapine, olanzapine, aripiprazole, and especially ziprasidone may be associated with a higher risk of seizures.

4. Atypical antipsychotic-related seizure risk may be associated with the 5-HT_1A_/D2 ratio, although prospective studies and clinical causality assessments are still needed.

## 1. Introduction

Antipsychotics (APs) remain a mainstay of treatment for schizophrenia and are also widely prescribed for bipolar disorder, major depressive disorder, anxiety disorders, and for managing psychotic symptoms in delirium and dementia [1, 2, 3]. However, because of their pharmacological properties, APs are associated with a broad spectrum of adverse events (AEs). These include extrapyramidal symptoms (EPS); metabolic disturbances such as weight gain, type 2 diabetes, and hyperprolactinemia; cardiovascular complications such as the corrected QT interval (QTc) prolongation; and sexual dysfunction [4]. Among these AEs, seizures are relatively uncommon yet clinically serious complications. They are characterized by spontaneous, recurrent episodes of neuronal hyperexcitability, often originating in specific brain regions. Seizures not only endanger patients’ physical health but also compromise medication adherence and substantially impair quality of life—such as restrictions on driving and access to certain jobs. The use of APs is associated with isolated, subclinical electroencephalographic (EEG) changes in approximately 7% of patients without a history of epilepsy, and with the emergence of clinical seizures in 0.5% to 1.2% of this patient population [5].

Atypical APs (AAPs) represent a newer generation of APs and are now widely prescribed worldwide. Compared with typical APs, AAPs are associated with a significantly lower incidence of EPS and minimal effects on prolactin levels [6]. However, emerging evidence suggests that AAPs may carry an elevated risk of seizures relative to typical APs [7]. Among AAPs, clozapine has the strongest evidence linking it to seizures induction, although reported incidence rates vary widely across studies ranging from 1% to 5% [8, 9]. Clinical registries reveal seizures incidence of 0.3%–0.9% for most other AAPs [5]. Early recognition of AAPs—related seizures is critical to reduce the risk of misdiagnosis and to enable timely, appropriate management. Moreover, identifying which AAPs are more likely to provoke seizures may provide valuable guidance for clinical decision-making and support individualized treatment strategies.

Nevertheless, the association between AAPs and seizures risk remains unresolved. Large, population-based investigations are lacking. Bloechliger *et al*. [10] analysed data from the UK Clinical Practice Research Datalink (1998–2013) and reported seizures incidence rates (per 10,000 person-years) of 48.8 for current quetiapine users, 25.9 for risperidone users, and 19.0 for olanzapine users in a cohort of 60,121 patients with schizophrenia, affective disorders, or dementia; however, clozapine exposure was not assessed in that study. Using Taiwan’s National Health Insurance Research Database, Wu *et al*. [11] evaluated 1-year risk of new-onset seizures among antipsychotic-naïve patients with schizophrenia or mood disorders and found that clozapine, haloperidol and thioridazine were associated with a two- to three-fold higher risk of seizures compared with risperidone. Other than these studies, most of the evidence addressing the relationship between the AAPs and risk of seizures is dominated by case reports and expert opinion, which offer limited ability to quantify comparative risk across drugs [12, 13, 14, 15, 16]. Paradoxically, randomized controlled trials (RCTs)—often regarded as the highest level of evidence—have not resolved the question: a systematic review of 314 RCTs found no statistically significant difference in seizures incidence between APs drugs (0.09%) and placebo (0.11%) [17].

Several methodological and contextual limitations restrict confidence in the existing literature. First, study findings are inconsistent and heterogeneous in design, outcome definition and follow-up duration. Second, much of the available data are relatively old (predominantly pre-2015) and derive from region-specific databases (e.g., UK, Germany, Taiwan), which constrains the temporal relevance and generalisability of the results. Third, drug coverage in prior studies is incomplete: safety evidence for more recently introduced AAPs (for example, lurasidone, paliperidone, ziprasidone) is sparse. Finally, most investigations focus on single drug or limited pairwise comparisons; there is a paucity of systematic evaluations across a broad panel of AAPs within a unified analytical framework, and data addressing the association between AAPs and specific seizures subtypes are notably scarce.

To address these gaps directly, we designed this study to leverage the U.S. Food and Drug Administration (FDA) Adverse Event Reporting System (FAERS, https://www.fda.gov/drugs/drug-approvals-and-databases/fda-adverse-event-reporting-system-faers-database), a global pharmacovigilance database that aggregates spontaneous reports from multiple countries and regions, thereby providing geographically diverse and extensive real-world evidence. Compared with previous studies, our analysis benefits from the most up-to-date FAERS dataset, encompassing reports through 2025 Q2, which substantially enhances the temporal relevance and comprehensiveness of the findings. We conducted a systematic pharmacovigilance evaluation of eight AAPs (clozapine, quetiapine, olanzapine, aripiprazole, ziprasidone, risperidone, lurasidone and paliperidone), applying four distinct disproportionality analysis algorithms within a unified analytical framework. This approach enables a robust and reliable real-world head-to-head comparison, ultimately generating a data-driven hierarchy of seizures risk among AAPs to better inform clinical decision-making and pharmacovigilance strategies.

## 2. Materials and Methods

### 2.1 Data Source

Data for this study were extracted from the FAERS database, which contains AE reports submitted on a voluntary basis in the United States by healthcare professionals (pharmacists, nurses, physicians) and consumers (patients, family members). FAERS is designed to monitor post-marketing drug safety. The FAERS dataset comprises seven data tables: “DEMO”, “DRUG”, “INDI”, “OUTC”, “REAC”, “RPSR”, and “THER”, each containing different types of information. Three tables, “DEMO”, “DRUG”, and “REAC” were used in the present analysis. “DEMO” includes demographic characteristics such as age, sex, and reporting country. “DRUG” provides detailed information on medications. “REAC” records AEs, identified by preferred terms (PTs) coded according to the Medical Dictionary for Regulatory Activities (MedDRA). In MedDRA, each PT is hierarchically organized and assigned to one or more corresponding high-level terms (HLTs), high-level group terms (HLGTs), and system organ class (SOC) categories [10]. In FAERS, each medication is classified by its suspected role in the reported AEs as primary suspect (PS), secondary suspect (SS), interacting (I), or concomitant (C).

### 2.2 Data Collection and Preprocessing

Data spanning the first quarter of 2004 through the second quarter of 2025 were downloaded from the FDA website in Extensible Markup Language (XML) and Comma-Separated Values (CSV) formats. These included records from both the Legacy Adverse Event Reporting System (LAERS) database (January 2004 to August 2012) and the FAERS database (September 2012 to date). Data standardization and deduplication primarily followed the workflow described by Banda *et al*. [18] using Structured Query Language (SQL) tools.

The main steps were as follows:

(1) Clean and standardize the downloaded data, including deduplication and normalization of drug names and AE outcomes, to ensure accuracy and consistency.

(2) Integrate LAERS and FAERS data, standardizing key field names (e.g., renaming isr and case to primaryid and caseid).

(3) Fill missing values using the DEMOyyQq table and deduplicate cases to avoid redundancy.

(4) Process drug data (DRUGyyQq table), mapping drug names to RxNorm concepts and enhancing integration using OHDSI standard concept identifiers (CUIs) and SNOMED-CT identifiers.

(5) Map drug names and AEs to standardized concepts in RxNorm, SNOMED-CT, and other controlled terminologies to ensure consistency and comparability.

(6) Merge LAERS and FAERS drug data into a single table, incorporating both historical and current case identifiers to maximize completeness.

**Fig. 1.**
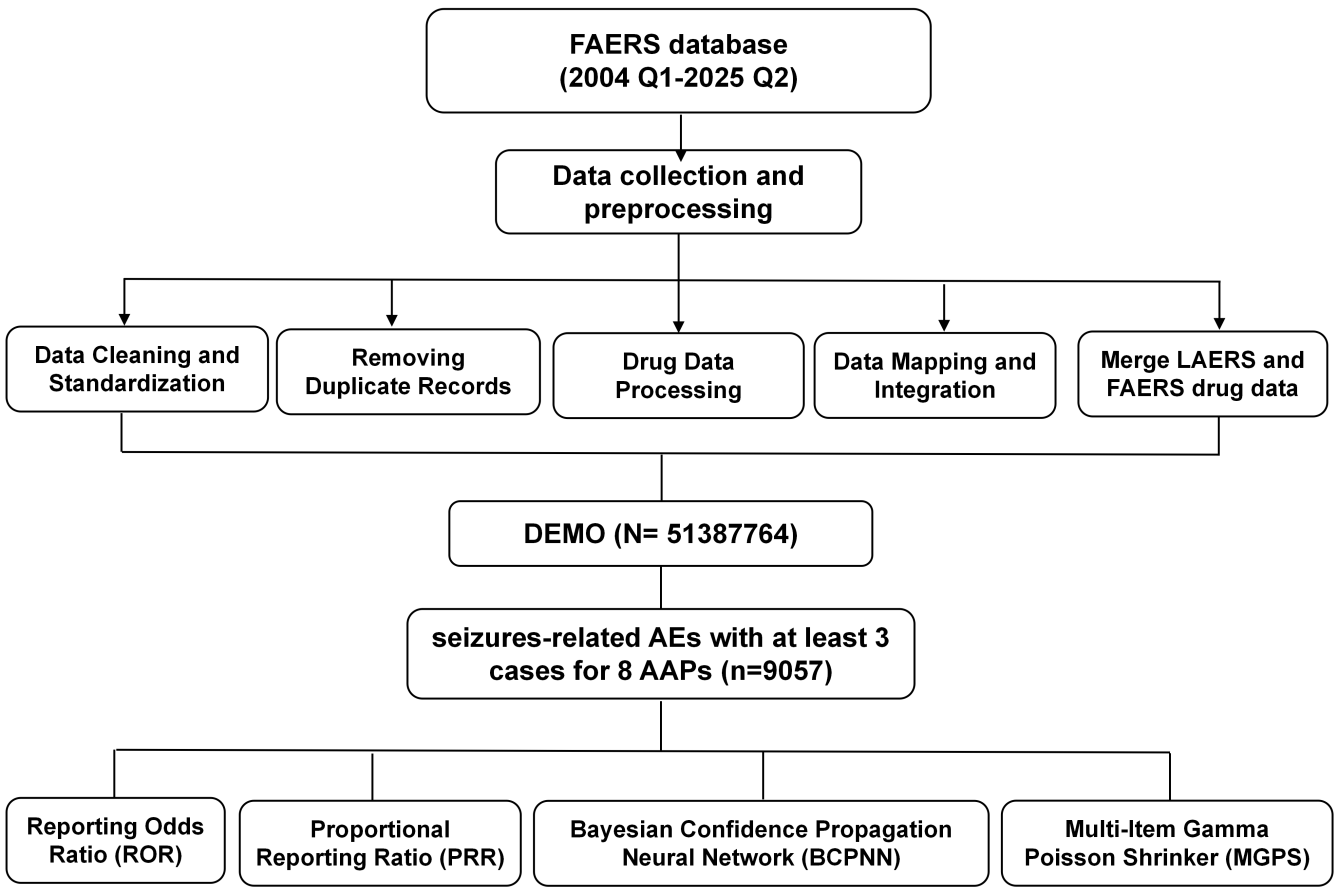
**A flowchart of the whole study**. FAERS, Food and Drug Administration (FDA) Adverse Event Reporting System; LAERS, Legacy Adverse Event Reporting System; DEMO, Demographic Information Screening; AEs, adverse events; AAPs, atypical antipsychotics.

This workflow (Fig. 1) produced a cleaned and standardized FAERS dataset, along with source code to support reproducibility and subsequent analyses. For this study, we focused on commonly prescribed AAPs previously reported to carry potential seizures risk [17]. Although lurasidone and ziprasidone are relatively newer agents, their increasing clinical use and limited evidence on seizures risk warranted their inclusion. Therefore, eight AAPs were analyzed: clozapine, olanzapine, aripiprazole, paliperidone, quetiapine, risperidone, ziprasidone, and lurasidone. These drugs were designated as PS, indicating that they were considered the most likely cause of seizures among all medications taken by each patient. To comprehensively capture seizures-related AEs, we extracted all reports containing PTs under the HLGT “Seizures (incl subtypes)” according to MedDRA® (version 25.1, the Medical Dictionary for Regulatory Activities terminology is the international medical terminology developed under the auspices of the International Council for Harmonisation of Technical Requirements for Pharmaceuticals for Human Use (ICH), Geneva, Switzerland).

### 2.3 Statistical Analysis

Disproportionality analysis was used to identify drug-related AE signals. Four algorithms were applied: reporting odds ratio (ROR), proportional reporting ratio (PRR), information component (IC) derived from the Bayesian Confidence Propagation Neural Network (BCPNN), and the empirical Bayesian geometric mean (EBGM) from the Multi-Item Gamma Poisson Shrinker (MGPS) [19, 20, 21]. Detailed formulas and signal-detection thresholds for these methods are provided in Table 1. ROR and PRR are effective for early signal detection but may generate false positives when the number of reports is small [22]. Bayesian methods (IC and EBGM) provide more stable estimates with sparse data. In this study, cases were defined as reports co-mentioning the targeted AAPs and seizures-related AEs. Only AEs with at least three reports were included in the final disproportionality analysis. Higher values in these methods indicate stronger associations between the drug and AE. Dichotomous variables were summarized as frequencies and percentages, while continuous variables were described as means with standard deviations. All analyses were performed using Microsoft Excel 2019 (Microsoft, Redmond, WA, USA), GraphPad Prism 9 (GraphPad, San Diego, CA, USA), and IBM SPSS Statistics 23 (IBM, Armonk, NY, USA).

**Table 1.** **Summary of four algorithms used for disproportionality analysis**.

Algorithms	Equation	Threshold
ROR	$R\,O\,R=\frac{a/c}{b/d}$	N ≥3, ROR_025_ >1
	$R\,O\,R_{95\%\,C\,I}=e^{\ln\left(R\,O\,R\right)\pm 1.96\,\sqrt{\left(\frac{1}{a}+\frac{1}{b}+\frac{1}{c}+\frac{1}{d}\right)}}$	
PRR	$P\,R\,R=\frac{a\,\left(c+d\right)}{c\,\left(a+b\right)}$	PRR ≥2, χ^2^ ≥4, N ≥3
	$\chi^{2}=\frac{\left[{\left(a\,d-b\,c\right)}^{2}\right]\,\left(a+b+c+d\right)}{\left[\left(a+b\right)\,\left(c+d\right)\,\left(a+c\right)\,\left(b+d\right)\right]}$	
BCPNN	$I\,C=\log_{2}\frac{a+0.5}{a_{\exp+0.5}}$	IC_025_ >0
	$a_{\text{exp }}=\frac{\left(a+b\right)*\left(a+c\right)}{\left(a+b+c+d\right)}$	
	$I\,C_{025}=I\,C-3.3*{\left(a+0.5\right)}^{-0.5}-2*{\left(a+0.5\right)}^{-1.5}$	
MGPS	$E\,B\,G\,M=\frac{a\,\left(a+b+c+d\right)}{\left(a+c\right)\,\left(a+b\right)}$	EBGM_05_ >2
	$95\%\,C\,I=e^{\mathrm{In}\left(\mathrm{EBGM}\right)\pm 1.96\,\sqrt{\left(\frac{1}{a}+\frac{1}{b}+\frac{1}{c}+\frac{1}{d}\right)}}$	

a is the number of AE cases attributable to the suspected drug; b is the number of AE cases attributable to other drugs; c is the number of other AE cases attributable to the suspected drug; d is the number of other AE cases attributable to other drugs. Abbreviations: χ^2^, chi-squared; ROR, reporting odds ratio; PRR, proportional reporting ratio; BCPNN, Bayesian Confidence Propagation Neural Network; MGPS, Multi-Item Gamma Poisson Shrinker; ROR_025_, the lower limit of the 95% CI of ROR; IC, information component; IC_025_, the lower limit of the 95% CI of IC; EBGM, empirical Bayesian geometric mean; EBGM_05_, the lower limit of the 95% CI of EBGM.

### 2.4 Correlation Analysis of Possible Mechanisms Underlying AAPs-Related Seizures

To explore potential pharmacological mechanisms of AAPs-associated seizures, Spearman correlation analysis was conducted. Binding affinity data (Ki values) for six major receptors (D1, D2, D3, 5-HT_1A_, 5-HT_2A_, and 5-HT_2C_) of the eight AAPs were obtained from the Psychoactive Drug Screening Program (PDSP) Ki Database (https://pdsp.unc.edu/). For receptors with multiple Ki values, the mean value was calculated by averaging all available measurements. The inverse of the mean Ki values (where larger values indicate stronger binding) was used as the affinity index. Three receptor ratios (5-HT_1A_/D2, 5-HT_2A_/D2, and 5-HT_2C_/D2) were then correlated with ROR values to examine potential pharmacodynamic relationships.

## 3. Results

**Fig. 2.**
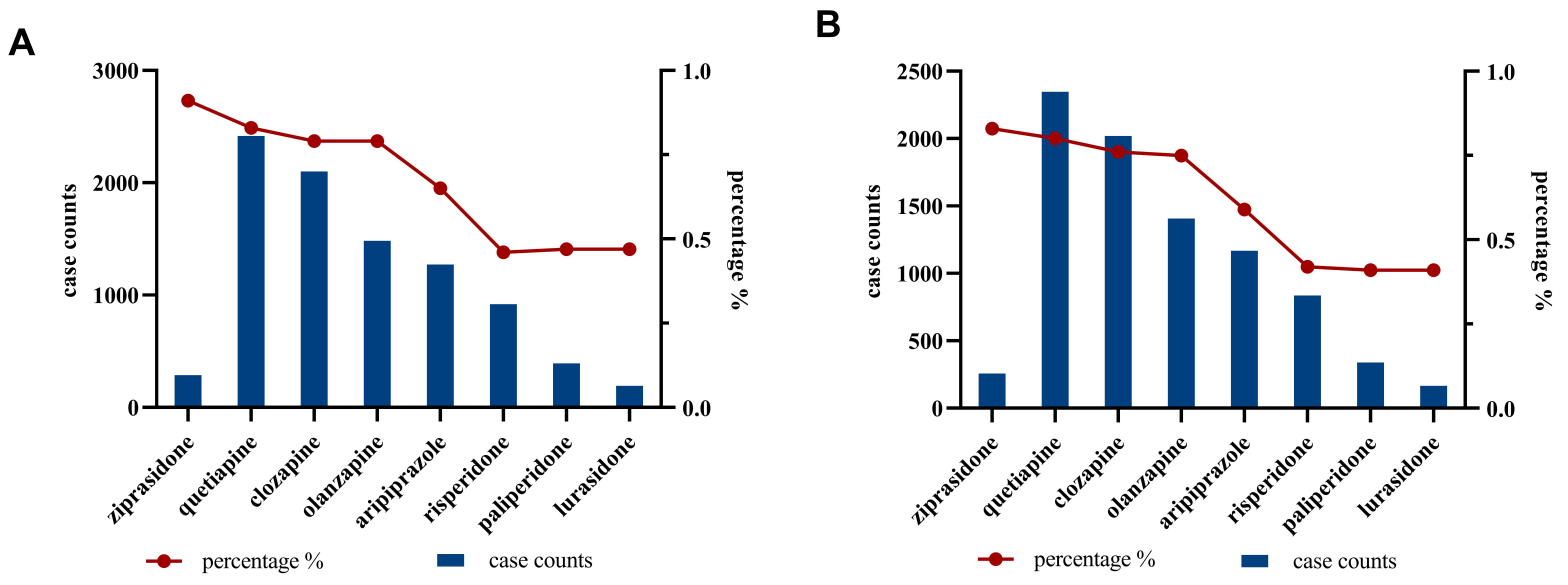
**The number and proportion of seizures-related AEs for the eight AAPs**. (A) The reported cases and percentage of seizures-related AEs in all reported cases for eight AAPs before ROR algorithm. (B) The reported cases and percentage of seizures-related AEs in all reported cases for eight AAPs after ROR algorithm (with at least 3 cases and ROR_025_ >1).

During the study period, a total of 51,387,764 cases were reported to FAERS. Reports involving AAPs ranged from 31,438 for ziprasidone to 292,829 for quetiapine. A total of 5767 PTs were submitted for clozapine, 5767 for quetiapine, 5262 for olanzapine, 4560 for aripiprazole, 2210 for lurasidone, 4335 for risperidone, 3064 for paliperidone, 2471 for ziprasidone. Of these, information of seizures-related AEs (all reported cases) for eight AAPs were as follows: 34 PTs and 2434 reported cases (0.83%) for quetiapine, 26 PTs and 2109 reported cases (0.79%) for clozapine, 25 PTs and 1493 reported cases (0.79%) for olanzapine, 24 PTs and 1283 reported cases (0.65%) for aripiprazole, 22 PTs and 931 reported cases (0.47%) for risperidone, 14 PTs and 291 reported cases (0.93%) for ziprasidone, 14 PTs and 198 reported cases (0.49%) for lurasidone, 17 PTs and 401 reported cases (0.48%) for paliperidone. The final analysis included 9057 reports of AAPs–seizures pairs (reported cases ≥3). The number and proportion of seizures-related AEs for the eight AAPs, before and after application of the ROR algorithm, are shown in Fig. 2.

### 3.1 Descriptive Analysis

Demographic characteristics of 8540 cases with significant seizures-related signals detected by ROR analysis at the PT level are summarized in Table 2. Sex-based differences were observed for several APs. Clozapine, risperidone and paliperidone had higher proportions of male cases (male-to-female ratios of 1.79, 1.80 and 1.92, respectively), whereas quetiapine, ziprasidone, and lurasidone showed higher proportions in females (female-to-male ratios of 1.37, 1.53, and 1.66, respectively). Olanzapine and aripiprazole demonstrated minimal sex-based differences. Regarding geographic distribution, the majority of reports for paliperidone, aripiprazole, ziprasidone, and lurasidone originated from the United States, representing over half of all cases.

**Table 2.** **The population characteristics for eight AAPs**.

Characteristics		Clozapine	Quetiapine	Olanzapine	Aripiprazole	Ziprasidone	Risperidone	Lurasidone	Paliperidone
Age (mean ± SD)		42.21 ± 15.41	40.36 ± 18.46	41.17 ± 19.43	35.83 ± 19.45	35.06 ± 15.86	38.65 ± 24.47	34.49 ± 16.00	39.68 ±17.15
Age group n (%)									
	≤18	65 (3.2%)	197 (8.4%)	122 (8.7%)	183 (15.7%)	23 (8.9%)	161 (19.3%)	13 (7.8%)	22 (6.5%)
	19–59	1212 (60.0%)	1179 (50.3%)	713 (50.6%)	493 (42.2%)	120 (46.5%)	303 (36.2%)	67 (40.4%)	183 (54.1%)
	≥60	208 (10.3%)	256 (10.9%)	186 (13.2%)	111 (9.5%)	7 (2.7%)	122 (14.6%)	6 (3.6%)	28 (8.3%)
	Unknown	535 (26.5%)	714 (30.4%)	387 (27.5%)	381 (32.6%)	108 (41.9%)	250 (29.9%)	80 (48.2%)	105 (31.1%)
Sex n (%)									
	Male	1220 (60.4%)	914 (39.0%)	637 (45.2%)	465 (39.8%)	87 (33.7%)	486 (58.1%)	53 (31.9%)	203 (60.1%)
	Female	682 (33.8%)	1249 (53.2%)	640 (45.5%)	539 (46.1%)	133 (51.6%)	270 (32.3%)	88 (53.0%)	106 (31.4%)
	Unknown	118 (5.8%)	183 (7.8%)	131 (9.3%)	164 (14.0%)	38 (14.7%)	80 (9.6%)	25 (15.1%)	29 (8.6%)
Reporting country n (%)									
	US	506 (25.0%)	1166 (49.7%)	395 (28.1%)	605 (51.8%)	176 (68.2%)	252 (30.1%)	146 (88.0%)	172 (50.9%)
	Non-US	1362 (67.4%)	1106 (47.1%)	875 (62.1%)	512 (43.8%)	34 (13.2%)	526 (62.9%)	18 (10.8%)	166 (49.1%)
	Unknown	152 (7.5%)	74 (3.2%)	138 (9.8%)	51 (4.4%)	48 (18.6%)	58 (6.9%)	2 (1.2%)	0 (0.0%)

### 3.2 Disproportionality Analysis

**Fig. 3.**
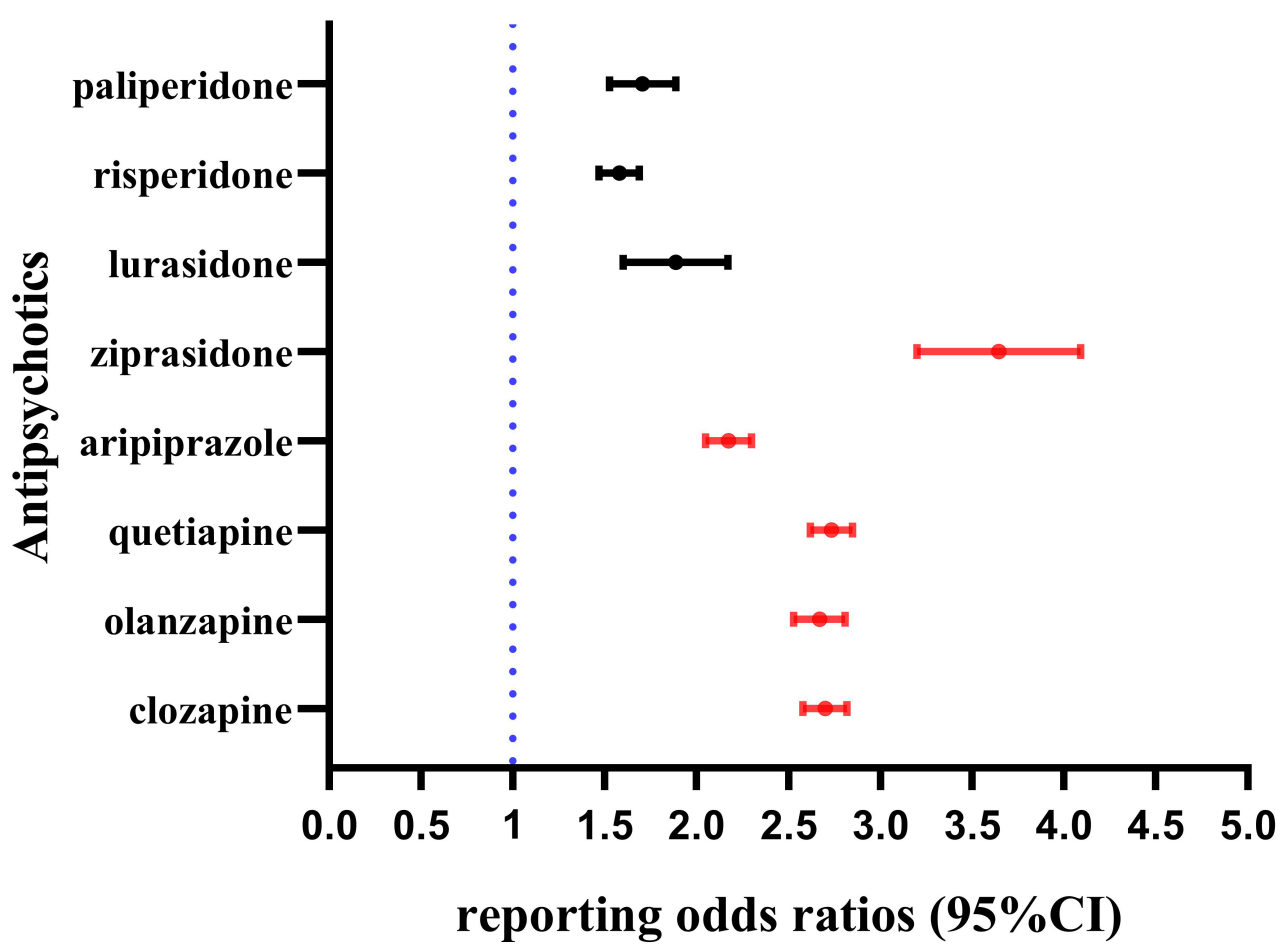
**The signal strength of seizures-related AEs for eight AAPs by ROR algorithm at HLGT level**.

The ROR algorithm was primarily used to identify significant signals. At the HLGT level, all of eight AAPs exhibited significant disproportionality, with ziprasidone showing the strongest association (ROR = 3.63, ROR_025_ = 3.21), followed by quetiapine (ROR = 2.73, ROR_025_ = 2.62), clozapine (ROR = 2.70, ROR_025_ = 2.58), olanzapine (ROR = 2.67, ROR_025_ = 2.53), and aripiprazole (ROR = 2.17, ROR_025_ = 2.05). Lurasidone (ROR = 1.87, ROR_025_ = 1.61), paliperidone (ROR = 1.70, ROR_025_ = 1.53), and risperidone (ROR = 1.58, ROR_025_ = 1.47) exhibited relatively lower signals than other AAPs (Fig. 3).

**Fig. 4.**
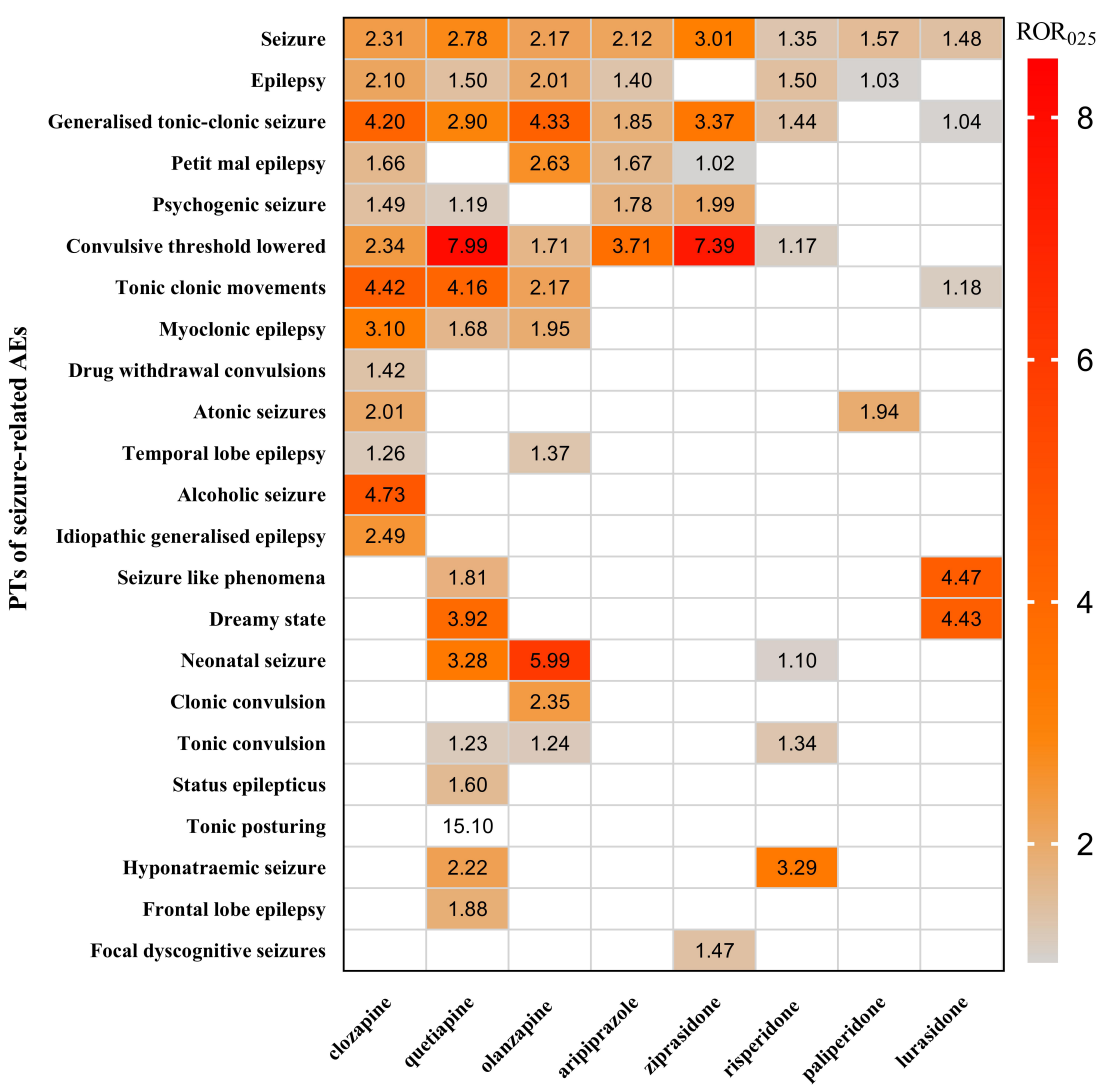
**A heat map of the signal strength of seizures-related AEs for eight AAPs by ROR algorithm at PT level**.

At the PT level, each AAP demonstrated a distinct seizures-related profile. Quetiapine was associated with the broadest spectrum, encompassing 15 PTs, followed by clozapine (13 PTs), olanzapine (11 PTs), risperidone (7 PTs), aripiprazole (6 PTs), ziprasidone (6 PTs), lurasidone (5 PTs), and paliperidone (3 PTs). The strongest PT-level signals for each AAP included clozapine–alcoholic seizure (n = 4; ROR = 13.02, PRR = 13.02), quetiapine–tonic posturing (n = 7; ROR = 33.93, PRR = 33.93), olanzapine–convulsion neonatal (n = 17; ROR = 9.72, PRR = 9.72), aripiprazole–convulsive threshold lowered (n = 9; ROR = 7.20, PRR = 7.20), ziprasidone–convulsive threshold lowered (n = 4; ROR = 19.80, PRR = 19.80), risperidone–hyponatremic seizure (n = 6; ROR = 7.41, PRR = 7.41), lurasidone–dreamy state (n = 3; ROR = 13.82, PRR = 13.82), and paliperidone–atonic seizures (n = 5; ROR = 4.68, PRR = 4.67) (Table 3 and Fig. 4).

**Table 3.** **The signal strength of seizures-related AEs profile for eight AAPs by four algorithms at PT level**.

Drugs	PT	Case number	ROR (95% CI)	PRR	χ ^2^	IC (IC_025_)	EBGM (EBGM_05_)
Clozapine	Seizure	1211	2.44 (2.31–2.58)	2.44	1013.63	1.27 (1.18)	2.42 (2.28)
	Generalised tonic-clonic seizure	330	4.69 (4.20–5.23)	4.68	933.74	2.19 (2.01)	4.60 (4.12)
	Epilepsy	305	2.35 (2.10–2.63)	2.35	232.97	1.22 (1.03)	2.33 (2.08)
	Petit mal epilepsy	49	2.20 (1.66–2.92)	2.20	31.75	1.11 (0.64)	2.19 (1.65)
	Tonic clonic movements	33	6.25 (4.42–8.84)	6.25	140.87	2.50 (1.92)	6.08 (4.30)
	Myoclonic epilepsy	29	4.48 (3.10–6.47)	4.48	76.58	2.06 (1.44)	4.40 (3.04)
	Drug withdrawal convulsions	19	2.24 (1.42–3.52)	2.24	12.88	1.11 (0.34)	2.22 (1.42)
	Atonic seizures	12	3.55 (2.01–6.28)	3.55	21.58	1.67 (0.69)	3.50 (1.98)
	Psychogenic seizure	11	2.70 (1.49–4.89)	2.70	11.58	1.32 (0.29)	2.67 (1.47)
	Convulsive threshold lowered	8	4.71 (2.34–9.50)	4.71	22.83	1.93 (0.72)	4.62 (2.29)
	Temporal lobe epilepsy	6	2.82 (1.26–6.31)	2.82	6.93	1.29 (–0.12)	2.79 (1.25)
	Alcoholic seizure	4	13.02 (4.73–35.83)	13.02	41.55	2.44 (0.68)	12.25 (4.45)
	Idiopathic generalised epilepsy	3	7.89 (2.49–25.03)	7.89	17.34	1.97 (–0.10)	7.62 (2.40)
Quetiapine	Seizure	1575	2.90 (2.76–3.05)	2.89	1918.65	1.51 (1.43)	2.86 (2.72)
	Generalised tonic-clonic seizure	256	3.29 (2.90–3.72)	3.28	399.47	1.69 (1.48)	3.24 (2.87)
	Epilepsy	244	1.70 (1.50–1.93)	1.70	70.03	0.76 (0.55)	1.70 (1.49)
	Status epilepticus	109	1.94 (1.60–2.34)	1.94	48.79	0.94 (0.62)	1.93 (1.59)
	Tonic clonic movements	34	5.86 (4.16–8.24)	5.86	132.48	2.42 (1.84)	5.70 (4.05)
	Convulsive threshold lowered	22	12.30 (7.99–18.96)	12.30	213.42	3.23 (2.51)	11.56 (7.50)
	Seizure like phenomena	21	2.78 (1.81–4.28)	2.78	23.60	1.40 (0.67)	2.75 (1.79)
	Myoclonic epilepsy	19	2.65 (1.68–4.16)	2.65	19.16	1.33 (0.56)	2.62 (1.67)
	Neonatal seizure	15	5.49 (3.28–9.17)	5.49	53.36	2.23 (1.36)	5.35 (3.20)
	Tonic convulsion	14	2.09 (1.23–3.54)	2.09	7.86	1.00 (0.10)	2.08 (1.23)
	Dreamy state	11	7.16 (3.92–13.09)	7.16	56.02	2.46 (1.44)	6.92 (3.79)
	Psychogenic seizure	10	2.23 (1.19–4.15)	2.23	6.66	1.06 (–0.01)	2.21 (1.18)
	Tonic posturing	7	33.93 (15.10–76.24)	33.93	187.29	3.33 (2.03)	28.57 (12.71)
	Hyponatraemic seizure	6	5.01 (2.22–11.28)	5.01	18.72	1.91 (0.50)	4.90 (2.18)
	Frontal lobe epilepsy	3	5.95 (1.88–18.80)	5.95	11.94	1.78 (–0.29)	5.79 (1.83)
Olanzapine	Seizure	820	2.33 (2.17–2.49)	2.32	613.62	1.21 (1.09)	2.31 (2.16)
	Generalised tonic-clonic seizure	246	4.91 (4.33–5.57)	4.91	752.12	2.26 (2.05)	4.84 (4.27)
	Epilepsy	212	2.30 (2.01–2.63)	2.30	154.25	1.19 (0.96)	2.29 (2.00)
	Petit mal epilepsy	54	3.44 (2.63–4.49)	3.43	92.02	1.74 (1.28)	3.40 (2.60)
	Convulsion neonatal	17	9.72 (5.99–15.76)	9.72	128.34	2.92 (2.11)	9.42 (5.80)
	Myoclonic epilepsy	15	3.24 (1.95–5.39)	3.24	22.95	1.58 (0.71)	3.21 (1.93)
	Tonic clonic movements	14	3.68 (2.17–6.24)	3.68	26.95	1.74 (0.84)	3.64 (2.15)
	Clonic convulsion	10	4.39 (2.35–8.21)	4.39	25.79	1.90 (0.83)	4.34 (2.32)
	Tonic convulsion	10	2.31 (1.24–4.31)	2.31	7.40	1.12 (0.04)	2.30 (1.24)
	Temporal lobe epilepsy	5	3.31 (1.37–8.00)	3.31	7.97	1.44 (–0.12)	3.28 (1.36)
	Convulsive threshold lowered	5	4.13 (1.71–9.98)	4.13	11.67	1.67 (0.11)	4.08 (1.69)
Aripiprazole	Seizure	836	2.27 (2.12–2.43)	2.27	587.85	1.17 (1.06)	2.26 (2.11)
	Epilepsy	158	1.64 (1.40–1.91)	1.64	38.82	0.70 (0.44)	1.63 (1.40)
	Generalised tonic-clonic seizure	117	2.21 (1.85–2.66)	2.21	77.17	1.13 (0.83)	2.20 (1.84)
	Petit mal epilepsy	38	2.30 (1.67–3.17)	2.30	27.81	1.17 (0.63)	2.29 (1.67)
	Psychogenic seizure	10	3.31 (1.78–6.18)	3.31	15.96	1.57 (0.49)	3.29 (1.76)
	Convulsive threshold lowered	9	7.20 (3.71–13.96)	7.20	46.72	2.42 (1.28)	7.03 (3.62)
Ziprasidone	Seizure	203	3.45 (3.01–3.97)	3.44	350.83	1.77 (1.54)	3.43 (2.99)
	Generalised tonic-clonic seizure	39	4.61 (3.37–6.32)	4.62	110.03	2.14 (1.60)	4.60 (3.36)
	Petit mal epilepsy	6	2.27 (1.02–5.06)	2.27	4.26	1.05 (–0.37)	2.27 (1.02)
	Convulsive threshold lowered	4	19.80 (7.39–53.08)	19.80	70.55	2.68 (0.91)	19.58 (7.30)
	Psychogenic seizure	3	6.20 (1.99–19.25)	6.20	13.02	1.83 (–0.24)	6.18 (1.99)
	Complex partial seizures	3	4.57 (1.47–14.19)	4.57	8.34	1.60 (–0.47)	4.56 (1.47)
Risperidone	Seizure	547	1.47 (1.35–1.60)	1.47	81.55	0.55 (0.41)	1.47 (1.35)
	Epilepsy	169	1.74 (1.50–2.02)	1.74	52.72	0.79 (0.54)	1.73 (1.49)
	Generalised tonic-clonic seizure	94	1.76 (1.44–2.16)	1.76	30.85	0.81 (0.47)	1.76 (1.44)
	Tonic convulsion	11	2.42 (1.34–4.39)	2.42	9.10	1.18 (0.16)	2.41 (1.33)
	Hyponatraemic seizure	6	7.41 (3.29–16.68)	7.41	32.34	2.29 (0.87)	7.23 (3.21)
	Convulsion neonatal	5	2.65 (1.10–6.40)	2.65	5.08	1.20 (–0.37)	2.63 (1.09)
	Convulsive threshold lowered	4	3.13 (1.17–8.39)	3.13	5.72	1.33 (–0.43)	3.10 (1.16)
Lurasidone	Seizure	133	1.75 (1.48–2.08)	1.75	42.91	0.80 (0.52)	1.75 (1.48)
	Generalised tonic-clonic seizure	18	1.65 (1.04–2.63)	1.65	4.65	0.70 (–0.09)	1.65 (1.04)
	Seizure like phenomena	9	8.61 (4.47–16.59)	8.61	60.12	2.61 (1.48)	8.56 (4.44)
	Tonic clonic movements	3	3.65 (1.18–11.35)	3.65	5.76	1.40 (–0.67)	3.65 (1.17)
	Dreamy state	3	13.82 (4.43–43.11)	13.82	35.29	2.28 (0.21)	13.68 (4.39)
Paliperidone	Seizure	278	1.77 (1.57–1.99)	1.77	92.83	0.82 (0.6)	1.77 (1.57)
	Epilepsy	55	1.34 (1.03–1.74)	1.34	4.70	0.42 (–0.03)	1.34 (1.03)
	Atonic seizures	5	4.68 (1.94–11.27)	4.67	14.33	1.80 (0.24)	4.65 (1.93)

Abbreviations: PT, preferred term; HLGT, high-level group term.

Because some AAPs showed large effect estimates despite small case counts, the BCPNN algorithm was applied to strengthen robustness. The largest BCPNN signals for each AAP were clozapine–generalised tonic-clonic seizure (n = 330; IC_025_ = 2.01), quetiapine–convulsive threshold lowered (n = 22; IC_025_ = 2.51), olanzapine–convulsion neonatal (n = 17; IC_025_ = 2.11), aripiprazole–convulsive threshold lowered (n = 9; IC_025_ = 1.28), lurasidone–seizure like phenomena (n = 9; IC_025_ = 1.48), paliperidone–seizure (n = 278; IC_025_ = 0.6), risperidone–hyponatraemic seizure (n = 6; IC_025_ = 0.87), and ziprasidone–generalised tonic-clonic seizure (n = 39; IC_025_ = 1.60) (Table 3).

### 3.3 Correlative Analysis Between ROR Signals and Receptor Affinity

Inverse Ki values of the D1, D2, D3, 5-HT_1A_, 5-HT_2A_, and 5-HT_2C_ receptors, as well as receptor ratios (5-HT_1A_/D2, 5-HT_2A_/D2, and 5-HT_2C_/D2), were incorporated into a Spearman correlation analysis with ROR_025_ values at the HLGT level across the eight AAPs. Spearman’s rank analysis revealed a significant negative correlation between seizure reporting and the 5-HT_1A_/D_2_ receptor ratio (rs = –0.79; *p* = 0.036). No significant correlations were observed for the other receptors or receptor ratios (*p* > 0.05) (Table 4).

**Table 4.** **Correlative analysis between ROR signal and receptor binding affinity (Ki) values**.

Antipsychotics	ROR_025_	D1	D2	D3	5-HT_1A_	5-HT_2A_	5-HT_2C_	5-HT_1A_/D2	5-HT_2A_/D2	5-HT_2C_/D2
Clozapine	2.58	240.18	157.55	307.45	139.48	10.30	49.53	0.89	0.07	0.31
Olanzapine	2.53	56.93	31.26	38.61	2124.71	4.58	17.86	67.97	0.15	0.57
Quetiapine	2.62	1057.85	500.22	386.44	328.25	345.38	2015.50	0.66	0.69	4.03
Aripiprazole	2.05	1173.50	2.01	4.60	12.28	32.95	50.34	6.11	16.39	25.04
Ziprasidone	3.21	97.28	4.57	7.32	18.44	0.61	3.34	4.04	0.13	0.73
Risperidone	1.47	313.44	4.31	6.66	425.26	0.76	28.57	98.67	0.18	6.63
Paliperidone	1.53	NA	NA	NA	404.00	0.91	38.00	NA	NA	NA
Lurasidone	1.61	NA	1.68	NA	29.90	19.00	481.00	17.80	11.31	286.31
Spearman’s Rho with	/	–0.31	0.68	0.60	–0.36	0.14	–0.05	–0.79	–0.50	–0.61
2-tailed (*p*-value)		(0.54)	(0.09)	(0.21)	(0.39)	(0.74)	(0.91)	(0.036)	(0.25)	(0.15)

Abbreviations: ROR_025_, the lower limit of the 95% CI of ROR. NA represents a missing Ki value.

## 4. Discussion

Several drug classes, including antidepressants, APs, and antibiotics, have been implicated in inducing seizures, which represent serious AEs of the central nervous system (CNS). Among patients receiving APs, concerns about seizures risk may adversely affect medication adherence and long-term outcomes. To our knowledge, this is the first pharmacovigilance–pharmacodynamic study using the real-world FAERS database to characterize seizures risk associated with eight AAPs.

Clozapine has long been considered the AP most strongly associated with seizures, demonstrating a well-established dose–response relationship: approximately 4.4% at high doses (>600 mg/day), 1.3% at moderate doses, and 1.0% at low doses (<300 mg/day). However, seizures rates reported in the literature vary widely, ranging from 4–6% to as high as 20% or even 22%, depending on the study design and time frame considered [8, 9, 23]. For example, an analysis of VigiBase (the WHO adverse drug reaction database) identified clozapine (9.0%) and quetiapine (5.9%) as the agents most frequently linked to convulsive AEs [24]. In our analysis, however, the proportion of clozapine-related seizures cases and corresponding signal values were not substantially higher than those for ziprasidone, quetiapine, or olanzapine, although they exceeded those for aripiprazole, risperidone, paliperidone, and lurasidone at the HLGT level.

Olanzapine and quetiapine, which share chemical similarity with clozapine, may also carry elevated seizures risk. In our analysis, quetiapine had the higher number of reports with seizures signals comparable to clozapine, whereas olanzapine showed fewer cases and lower signal values. Prior studies have reported similar seizures rates of about 0.9% for both agents [25, 26], consistent with our findings. Notably, ziprasidone, although relatively newer to the market, exhibited seizures signal values exceeded clozapine, quetiapine and olanzapine despite a smaller case count, highlighting the need for further study.

Aripiprazole has generally been regarded as one of the safer APs, with a reported seizures incidence of 0.1% [26]. Bloechliger *et al*. [10] reported that aripiprazole and risperidone were associated with the lowest seizures risk among AAPs. In an animal study, however, aripiprazole produced a proconvulsant rebound effect in genetically epilepsy-prone rats [27]. In our analysis, aripiprazole showed higher seizures signal values than risperidone, paliperidone, and lurasidone, though slightly lower than other AAPs. While aripiprazole is generally considered to have a favorable safety profile, emerging evidence-including a pharmacovigilance study identifying it as the only AP among 15 agents with a significant signal for obsessive-compulsive disorder/obsessive–compulsive symptoms (OCD/OCS) [28], suggests its AE spectrum may be broader than previously assumed.

Evidence for risperidone-associated seizures is limited; one population-based study reported an incidence of 24.1 per 10,000 person-years [10]. Data on paliperidone and lurasidone are even more sparse, and rigorous comparative evaluations of seizures risk for these agents remain lacking. In our FAERS-based pharmacovigilance analysis, risperidone, paliperidone and lurasidone consistently produced lower disproportionality signals for seizures than the other five AAPs, and each was associated with a comparatively narrow range of seizure-specific PTs.

At the PT level, all eight AAPs showed associations with particular seizures subtypes when assessed using the ROR algorithm. Quetiapine exhibited the broadest spectrum of seizure-related PTs. Notably, seven of the eight AAPs (except paliperidone) were linked to generalized tonic-clonic seizures in our dataset. Quetiapine was additionally associated with frontal-lobe epilepsy and status epilepticus-epilepsy phenotypes that may predispose to seizure clustering. Seizure clusters, defined as multiple acute seizures occurring within a short interval, are clinically important because they increase healthcare utilization and substantially worsen quality of life for patients and caregivers [29]. These findings suggested that by recognizing the associations between specific AAPs and seizure types, healthcare professionals can take appropriate precautions, monitor patients closely, and tailor treatment plans to minimize the risk of seizure clusters.

Seizures are traditionally described as resulting from an imbalance between excitatory and inhibitory neurotransmission. Excitatory neurotransmitters such as α-amino-3-hydroxy-5-methyl-4-isoxazolepropionic acid (AMPA), N-methyl-D-aspartate (NMDA), and kainate receptors promote neuronal activity, whereas inhibitory neurotransmitters such as GABA and glycine restrain excitability [30]. Clozapine, for instance, has been shown to potentiate neuronal activity by promoting glial release of D-serine, a co-agonist of NMDA receptors [31]. Increasing evidence suggests that multiple neuromodulatory systems contribute to epileptogenesis. Within the dopaminergic system, seizures modulation depends on receptor subtype and brain region, with animal studies consistently demonstrating opposing roles of D1-like and D2-like receptors in limbic seizures [32, 33]. Because most AAPs act as weak D2 antagonists while also targeting D1, D3, and serotonergic receptors (5-HT_1A_, 5-HT_2A_, 5-HT_2C_), receptor-binding profiles and 5-HT_2A_/D2 and 5-HT_2C_/D2 ratios are thought to influence both efficacy and adverse effects [34]. In the present analysis, Spearman’s correlation revealed a significant association between higher 5-HT_1A_/D2 affinity ratios and seizures-related AEs, supporting a role for serotonergic-dopaminergic interactions in mediating seizures risk.

Aringhieri *et al*. [34] classified AAPs into three categories based on receptor-binding profiles: risperidone as Level I with the narrowest spectrum, clozapine as Level III with the broadest spectrum, and all other AAPs as Level II with intermediate profiles. Aripiprazole stands apart for its unique “adaptive” pharmacological activity, acting as a full antagonist, moderate antagonist, or partial agonist at D2 receptors depending on dopamine levels and signaling context, consistent with biased ligand activity [35]. Paliperidone, the active metabolite of risperidone, shares similar pharmacology, while lurasidone displays comparable 5-HT_2C_/D2 affinity ratios to risperidone. Our results provided clinical validation for the receptor-based classification of AAPs. Clozapine, olanzapine, quetiapine and ziprasidone (Level II/III agents with broad binding profiles) demonstrated relatively higher and diverse seizures risk. Conversely, risperidone, paliperidone, and lurasidone (Level I/narrower-profile agents) formed a distinct low-risk cluster. Aripiprazole’s unique partial agonist mechanism may underpin its intermediate risk.

Hyponatremia has also been implicated in AAPs-associated seizures. APs can induce water intoxication, potentially leading to hyponatremia, which is often considered a manifestation of the syndrome of inappropriate antidiuretic hormone secretion (SIADH) [36]. Several case reports describe seizures associated with drug-induced hyponatremia in patients receiving quetiapine, olanzapine, ziprasidone, or risperidone [37, 38, 39, 40, 41]. The mechanism of AAPs-induced SIADH remains unclear, but it has been hypothesized that APs may stimulate antidiuretic hormone release, enhance its renal action, or induce dopamine receptor supersensitivity through chronic D2 blockade, thereby contributing to elevated hormone levels [42].

It is important to emphasize that correlation analyses indicate potential associations but do not establish causation. Seizures occurrence in patients taking AAPs can be influenced by multiple factors, including somatic conditions (e.g., prior seizures, brain injury, learning disabilities, dementia), pharmacological factors (e.g., dose titration, metabolism, drug–drug interactions, concomitant medications that lower seizures threshold), and study design differences, all of which may contribute to variability in observed outcomes [43]. Also, additional associations were found for younger age, APs polypharmacy, concomitant use of lithium [9], and patients with a history of convulsive disorder have also been reported to be more likely to have seizures [44].

### Limitations

Several limitations inherent to FAERS and spontaneous reporting systems constrain the interpretation of our results. First, FAERS comprises voluntary reports and is subject to well-documented reporting biases (under-reporting, stimulated reporting, and differential reporting by drug notoriety). Such biases may exaggerate the relative signal for drugs with an established reputation for seizures (for example, clozapine) and attenuate signals for newer or less-reported agents (for example, lurasidone or ziprasidone). Second, disproportionality metrics (ROR, PRR, BCPNN, MGPS) detect statistical associations in reporting frequency and should be interpreted as hypothesis-generating signals rather than unbiased estimates of incidence or relative risk. Third, FAERS lacks clinical granularity: critical confounders such as exact dose, duration of therapy, therapeutic monitoring, co-morbid medical and psychiatric diagnoses, concomitant medications that lower seizure threshold, and electroencephalographic or neuroimaging data are generally unavailable, precluding robust adjustment for these factors and preventing reliable dose–response or time-to-event analyses. Fourth, the absence of a well-defined denominator, the potential for duplicate or incomplete reports, and variable report quality are intrinsic limitations despite our application of rigorous data-cleaning procedures; these issues may affect the stability and precision of signal estimates. Finally, our analysis was limited to eight AAPs and therefore may not generalize to all APs.

Despite these limitations, several aspects of our study strengthen the credibility of the findings. We leveraged a large, contemporary FAERS extract (through 2025) with broad geographic representation, increasing the temporal relevance and global generalisability of the pharmacovigilance signals compared with many prior, region-restricted analyses. We applied four complementary disproportionality algorithms within a harmonized analytic framework, and we reported internal head-to-head comparisons across eight AAPs, which reduces some sources of analytic heterogeneity common to literature synthesis. The convergence of consistent signals across multiple methodologies and the biologic plausibility of the observed hierarchy of seizures signals lend weight to the results while still recognizing the observational and hypothesis-generating nature of the evidence.

Clinically, our results underscore the need for individualized risk assessment and cautious prescribing. Where possible, clinicians should employ the lowest effective dose and adopt gradual titration schedules, particularly in patients with predisposing factors for seizures. Polypharmacy with multiple APs, concomitant use of lithium, and co-prescription of other agents known to lower the seizure threshold merit careful reconsideration and close monitoring. Our PT-level analyses suggested that some agents are associated with a broader and potentially more severe spectrum of seizures phenotypes (for example, quetiapine was linked to a wider range of seizure-related PTs including frontal-lobe epilepsy and status epilepticus). Clinicians should therefore maintain heightened vigilance for clustering or focal seizure features that can precede generalized tonic-clonic events and may substantially increase healthcare utilization and patient/caregiver burden.

In summary, while spontaneous-report data cannot establish causality, our systematic, multi-method pharmacovigilance assessment of FAERS provides a contemporary, globally informed signal hierarchy that can inform clinical vigilance.

## 5. Conclusions

This pharmacovigilance study used FAERS data to characterize the spectrum and features of AAPs-related seizures. The findings indicated that clozapine, quetiapine, olanzapine, aripiprazole, and ziprasidone are associated with elevated seizures risk, although differences among these drugs were modest. The study also suggests that dopamine and serotonin receptor ratios may contribute to AAPs-related seizures risk. These results underscore the importance of careful monitoring and management of seizures risk in patients receiving AAPs therapy. Further prospective studies are needed to guide optimal clinical interventions and treatment strategies.

## Availability of Data and Materials

The data used in this study are publicly available from the Food and Drug Administration (FDA) Adverse Event Reporting System (FAERS, https://www.fda.gov/drugs/drug-approvals-and-databases/fda-adverse-event-reporting-system-faers-database) database.
